# A time-in-motion study of screening, brief intervention, and referral to treatment (SBIRT) implementation in healthcare settings

**DOI:** 10.1186/1940-0640-8-S1-A19

**Published:** 2013-09-04

**Authors:** Alexander J Cowell, William N Dowd, Justin Landwehr, Jeremy W Bray

**Affiliations:** 1 Behavioral Health and Criminal Justice Research Division, RTI International, RTP, NC, USA; 2 Behavioral Health and Criminal Justice Research Division, RTI International, Rockville, MD, USA

## Introduction

Between 2004 and 2013, the Substance Abuse and Mental Health Services Administration (SAMHSA) funded six cohorts of grantees to implement screening, brief intervention, and referral to treatment (SBIRT) services in selected sites. This paper presents data from the cross-site evaluation of the third cohort of state/tribal grantees on how staff allocate their time performing SBIRT activities (e.g. a screen). Because SBIRT activities such as screens may take little time to perform, data that rely on practitioner report to obtain such data may be inaccurate. Estimates gathered by observing practitioners’ time are likely to be more accurate.

## Methods

Participants were SBIRT practitioners from select sites within the four grantee organizations. Practitioners were observed in their duties over complete shifts. Using structured forms, observers recorded the time practitioners spent performing each of 18 pre-determined activities. We computed patient-level statistics for each service delivery component, as well as for patient-specific support and Government Performance and Results Act (GPRA) activities. We calculated shift-level statistics for SBIRT service delivery, patient support, general support, GPRA, non-SBIRT service delivery, and idle time.

## Results

Over 225 hours was recorded. The mean (SD) time to deliver a screen (n=99) was 4:38 minutes (4:04), and a brief intervention (n=68) took 6:51 (5:07). Practitioners spent an average of 7:00 (8:24) on activities supporting service delivery to a specific patient. At the shift level, practitioners spent 12% of their time delivering services, and 38% on support activities (general and patient-specific). Another 14% was spent waiting to engage available patients.

## Conclusions

SBIRT practitioners spend a relatively small portion of their time delivering services to patients. Organizations implementing SBIRT will need to realistically budget practitioner time spent face to face with patients.

